# A genome-wide association study of limb bone length using a Large White × Minzhu intercross population

**DOI:** 10.1186/s12711-014-0056-6

**Published:** 2014-11-04

**Authors:** Long-Chao Zhang, Na Li, Xin Liu, Jing Liang, Hua Yan, Ke-Bin Zhao, Lei Pu, Hui-Bi Shi, Yue-Bo Zhang, Li-Gang Wang, Li-Xian Wang

**Affiliations:** Key Laboratory of Farm Animal Genetic Resources and Germplasm Innovation of Ministry of Agriculture, Institute of Animal Science, Chinese Academy of Agricultural Sciences, 100193 Beijing, China; Jilin Academy of Agricultural Sciences, 130033 Changchun, China

## Abstract

**Background:**

In pig, limb bone length influences ham yield and body height to a great extent and has important economic implications for pig industry. In this study, an intercross population was constructed between the indigenous Chinese Minzhu pig breed and the western commercial Large White pig breed to examine the genetic basis for variation in limb bone length. The aim of this study was to detect potential genetic variants associated with porcine limb bone length.

**Methods:**

A total of 571 F2 individuals from a Large White and Minzhu intercross population were genotyped using the Illumina PorcineSNP60K Beadchip, and phenotyped for femur length (FL), humerus length (HL), hipbone length (HIPL), scapula length (SL), tibia length (TL), and ulna length (UL). A genome-wide association study was performed by applying the previously reported approach of genome-wide rapid association using mixed model and regression. Statistical significance of the associations was based on Bonferroni-corrected *P*-values.

**Results:**

A total of 39 significant SNPs were mapped to a 11.93 Mb long region on pig chromosome 7 (SSC7). Linkage analysis of these significant SNPs revealed three haplotype blocks of 495 kb, 376 kb and 492 kb, respectively, in the 11.93 Mb region. Annotation based on the pig reference genome identified 15 genes that were located near or contained the significant SNPs in these linkage disequilibrium intervals. Conditioned analysis revealed that four SNPs, one on SSC2 and three on SSC4, showed significant associations with SL and HL, respectively.

**Conclusions:**

Analysis of the 15 annotated genes that were identified in these three haplotype blocks indicated that *HMGA1* and *PPARD,* which are expressed in limbs and influence chondrocyte cell growth and differentiation, could be considered as relevant biological candidates for limb bone length in pig, with potential applications in breeding programs. Our results may also be useful for the study of the mechanisms that underlie human limb length and body height.

**Electronic supplementary material:**

The online version of this article (doi:10.1186/s12711-014-0056-6) contains supplementary material, which is available to authorized users.

## Background

In humans and other mammalian species, individuals with greater limb length have greater body height. In humans, limb bone length is highly correlated with body height, with correlation coefficients reaching 0.60 to 0.88 [1-3]. Thus, analysis of genetic variants associated with limb bone length can contribute to our understanding of human height. The ease with which experimental designs can be conducted in domestic pig makes this species an important large animal model for human research as well as a major farm animal for meat production [4]. In pigs, limb length influences body height and thus, ham yield, to a great extent [5]. Porcine cured ham, such as Jinhua ham and Iberian ham, is one of the most popular and valuable meat products worldwide. However, in China, ham products are produced from indigenous pig breeds, most of which have smaller limb lengths than western commercial pig breeds such as Large White, Landrace, and Duroc [6]. Thus, understanding the genetic basis of the variation in limb length between the two types of pig breeds is of economic importance.

Based on the high-density panels of single nucleotide polymorphisms (SNP), genome-wide association studies (GWAS) have been developed to identify DNA variants associated with many complex diseases and traits in humans and animals [7,8]. Although quantitative trait loci (QTL) that affect limb bone length have been detected on pig chromosomes (SSC for *Sus scrofa*) SSC1, 2, 3, 4, 5, 7, 14, 15, 16, 17 and X [9,10], to date, no GWAS has been carried out for this trait. The aim of this study was to detect potential genetic variants associated with limb bone length in a Large White × Minzhu intercross population using a GWAS and to identify candidate genes that are near these polymorphisms or that include them with major effects on this trait.

## Methods

### Ethics statement

All animals used in this study were treated according to the guidelines for experimental animals established by the Council of China. Animal experiments were approved by the Science Research Department of the Institute of Animal Science, Chinese Academy of Agricultural Sciences (Beijing, China).

### Population used and phenotype recording

An intercross population was developed between a western commercial pig breed (long-limbed Large White) and a Chinese indigenous pig breed (short-limbed Minzhu), as described previously [11]. A total of 571 F2 animals (53 litters) were obtained from 36 F1 dams, which were mated to nine sires. All F2 piglets were weaned at 35 days of age and male pigs were castrated three days after birth. All F2 animals were slaughtered at 240 ± 7 days in 30 batches (slaughter groups).

After slaughter, both the forelimb and hind limb were removed from the left side of the carcass of the F2 animals. Six limb bones were dissected from these limbs and their lengths were measured using a caliper according to Mao et al. [9]. These included the femur (total length from the greater trochanter to the intercondyloid fossa), humerus (total length from the head to the trochlea), hipbone (length from the crista iliaca to the ramus inferior ossis pubis), scapula (the maximum straight line distance from the cavitas glenoidalis to the border of the scapular cartilage), tibia (length from the intercondylar eminence to the medial malleolus), and ulna (length from the olecranon process to the styloid process). A normal distribution test was applied for each trait using the univariate normal procedure in SAS software.

### Genotyping and quality control

A total of 455 blood samples and 116 ear tissue samples were used for genomic DNA extraction using the salting-out method [12]. Genotyping was performed using Illumina PorcineSNP60 Genotyping BeadChip technology, which contained 62 163 SNPs across the whole genome. Quality control procedures were performed for the F2 individuals using the GenABEL package [13] within the R statistical environment. Quality control filtering of SNPs was done as follows: SNPs that had a call rate below 90%, or a minor allele frequency (MAF) below 3%, or that were not in Hardy-Weinberg equilibrium (HWE) with a *P*-value below 10^−6^ were eliminated. The final dataset that passed the quality control procedure and was used in the analysis contained 48 238 SNPs and 564 F2 individuals.

### Genome-wide association study

The GWAS was performed by applying the genome-wide rapid association approach using mixed model and regression [13,14], according to [11]. Sex, parity, and batch were selected as fixed effects for individuals and the litter effect was considered as a random effect. The DMU [15] and GenABEL software packages [13] in the R Language and Environment for Statistical Computing were used to analyze the data. A genome-wide significance threshold of 2.07E-08 (0.001/48238) was determined by the Bonferroni method, in which the conventional *P*-value was divided by the number of tests performed [16]. The most significant SNP was considered as a fixed effect and conditioned analysis was performed according to the GWAS procedure described above.

### Linkage disequilibrium analysis

Linkage disequilibrium (LD) analysis was performed on the region that contained all SNPs that were significantly associated with limb bone length. The Haploview v4.1 program [17] was used to calculate linkage disequilibrium measures and to visualize haplotype blocks.

## Results

### Phenotypes and correlations between traits

Descriptive statistics including means, standard deviations, minimum and maximum lengths of limb bones of the F2 individuals are in Table 1. Mean values for femur length (FL), humerus length (HL), hipbone length (HIPL), scapula length (SL), tibia length (TL), and ulna length (UL) were equal to 21.64 cm, 18.72 cm, 25.46 cm, 17.37 cm, 19.38 cm, and 21.04 cm, respectively. The normal distribution test showed that not all traits followed a normal distribution. However, residuals, which were estimated in step 1 of the GWAS protocol as described previously [11], followed a normal distribution and were used as the dependent trait to test the associations using a single locus regression analysis (data not shown). The correlation coefficients between the traits are in Table 2. All correlations were highly significant (*P* < 0.0001) and positive, and the coefficients ranged from 0.6211 to 0.7846.

**Table 1:** **Descriptive statistics of the length of six limb bones**

**Trait** ^**1**^	**Mean (cm)**	**Std**	**Minimum (cm)**	**Maximum (cm)**
SL	17.37	0.95	14.10	21.00
HL	18.72	1.32	15.00	22.80
UL	21.04	1.59	16.90	26.00
HIPL	25.46	2.14	15.10	31.50
FL	21.64	1.43	8.80	27.00
TL	19.38	1.48	15.80	27.70

^1^SL, scapula length; HL, humerus length; UL, ulna length; HIPL, hipbone length; FL, femur length; TL, tibia length.

**Table 2:** **Phenotypic correlation coefficients between limb bone lengths**

**Trait** ^**1**^	**HL**	**UL**	**HIPL**	**FL**	**TL**
SL	0.7358	0.7198	0.6373	0.7225	0.6211
HL		0.7846	0.6654	0.7573	0.7540
UL			0.6621	0.7820	0.7350
HIPL				0.6755	0.6233
FL					0.7236

^1^SL, scapula length; HL, humerus length; UL, ulna length; HIPL, hipbone length; FL, femur length; TL, tibia length. The correlation coefficients are listed in the upper triangle, and all P values of the correlation coefficients are lower than 0.0001.

### Genome-wide association study

**Figure 1:**
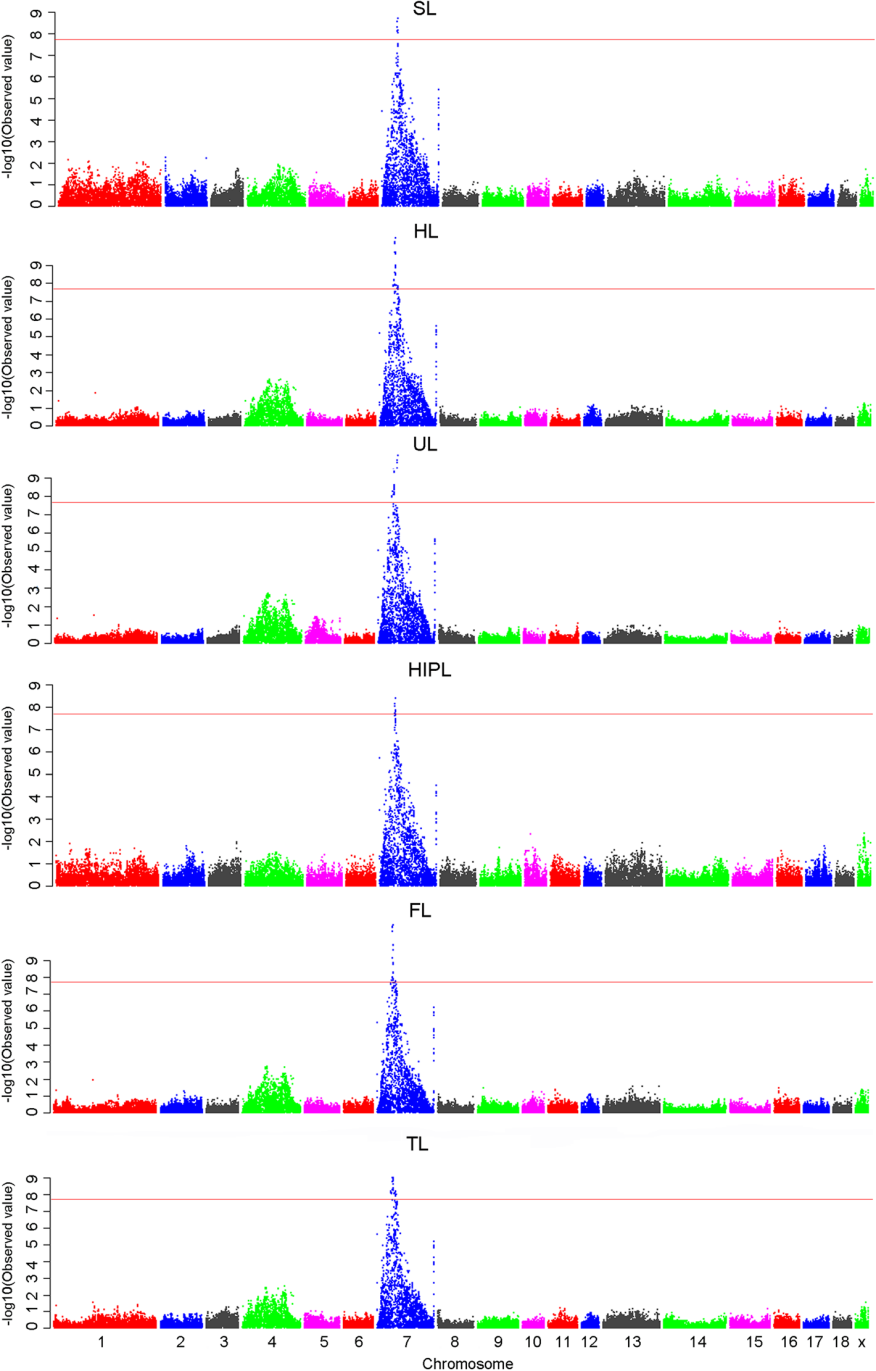
**Manhattan plots of genome-wide association study with lengths of six limb bones.** Chromosomes 1-18, X and Y are shown in different colors. The red horizontal line indicates the genome-wide significance level (-log_10_ (2.07E-08)).

**Figure 2:**
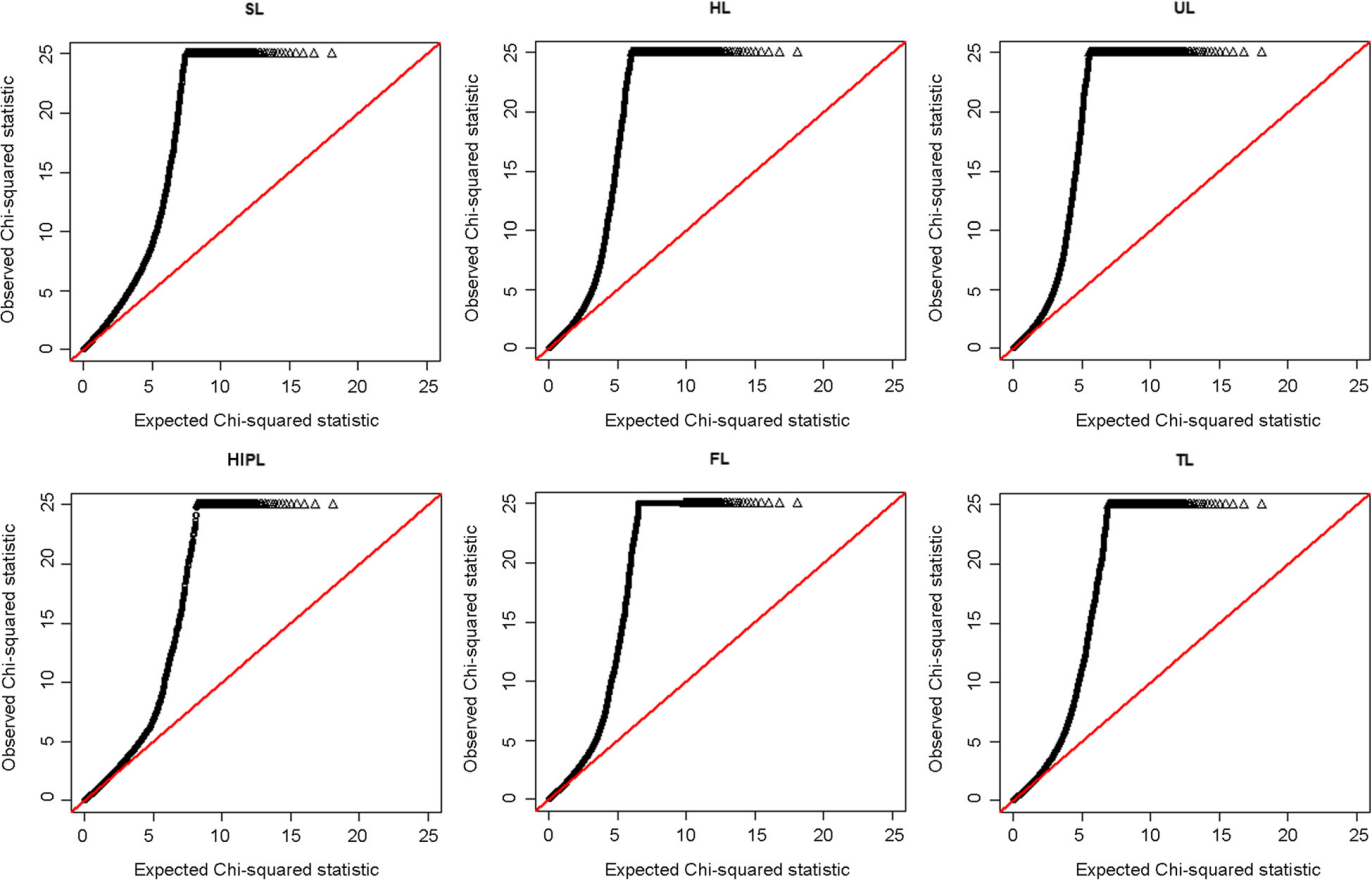
**Quantile-quantile (Q-Q) plots.** Results are shown in black. SNPs for which the test statistic exceeds 25 are represented by triangles.

After quality control, 48 238 SNPs and 564 F2 individuals were used for the GWAS. The selected SNPs were distributed over 18 autosomes and the X/Y chromosomes, as shown in [See Additional file 1: Table S1]. A threshold of 2.07E-08 (0.001/48238) was used in this study. Six traits were assessed; the resulting Manhattan plots and quantile-quantile (Q-Q) plots are in Figures 1 and 2, respectively. The Q-Q plot results showed an obvious deviation between the real and expected data and indicated that the association detected on pig chromosome SSC7 (SSC *Sus scrofa*) is statistically significant.

A total of 39 genome-wide significant SNPs were located in a 11.93 Mb (Megabase) long region of the long arm of SSC7 (i.e. SSC7q) between 31.24 and 43.17 Mb. Of the 39 SNPs, 29, 35, 12, 6, 39, and 27 were significantly associated with FL, HL, HIPL, SL, TL, and UL, respectively [See Additional file 2: Table S2]. All significant SNPs were used for BLAST searches on the pig genome database (Build 10.2) and 14 of the SNPs were located within 13 annotated genes. All other SNPs were between 4327 and 137 056 bp away from the nearest known genes. The most significant SNP for each trait was MARC0033464, which accounted for 47.93%, 57.89%, 52.28%, 37.50%, 51.97%, and 57.80% of the phenotypic variance for FL, HL, HIPL, SL, TL, and UL, respectively. In addition, 11 chromosome-wide significant (*P* < 6.16E-05) SNPs were identified in the distal region of SSC7q [See Additional file 3: Table S3].

### Conditioned analysis

A conditioned analysis was performed using the most significant SNP, MARC0033464, as a fixed effect. The Manhattan and Q-Q plots obtained from the conditioned analysis are in [See Additional file 4: Figure S1 and Additional file 5: Figure S2], respectively. No significant SNP was detected on SSC7 after the conditioned analysis. However, four SNPs, one on SSC2 and three on SSC4, showed chromosome-wide associations with SL and HL, respectively [See Additional file 6: Table S4].

### Haplotype analysis

**Figure 3:**
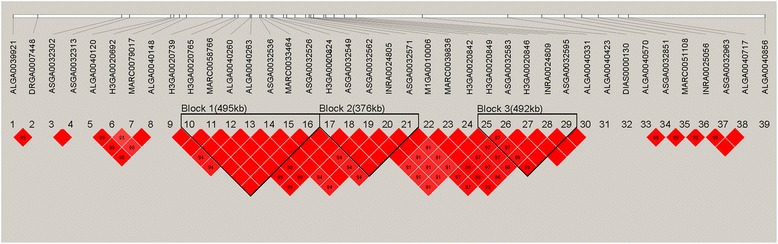
**Haplotype blocks in linkage disequilibrium (LD) in the 11.93 Mb region of SSC7 that contains all the significant SNPs.** Solid lines mark the three identified blocks. Block1 495 kb long that contains the most significant SNP i.e. MARC0033464 was in complete linkage disequilibrium (LD, r^2^ = 1).

Linkage analysis of the 11.93 Mb region identified three haplotype blocks that ranged in size from 376 kb to 495 kb (Figure 3). Block1 495 kb long (between H3GA0020765 and ASGA0032526) and block2 376 kb long (between H3GA0020824 and ASGA0032571) were both in complete LD (r^2^ = 1). Block1 contained the most significant SNP, MARC0033464, and six annotated genes, namely, *glutamate receptor, metabotropic 4* (*GRM4*); *high mobility group AT-hook 1* (*HMGA1*); *nudix (nucleoside diphosphate linked moiety X)-type motif 3* (*NUDT3*); *ribosomal protein S10* (*RPS10*); *SAM pointed domain containing ETS transcription factor* (*SPDEF*); and *protein kinase C and casein kinase substrate in neurons 1* (*PACSIN1*). Block2 contained five annotated genes, namely, *chromosome 6 open reading frame 106 ortholog* (*C6ORF106*), *small nuclear ribonucleoprotein polypeptide C* (*SNRPC*), *UHRF1 binding protein 1* (*UHRFBP1*), *TAF11 RNA polymerase II, TATA box binding protein (TBP)-associated factor* (*TAF11*), and *ankyrin repeat and sterile alpha motif domain containing 1A* (*ANKS1A*). Block 3 492 kb long (between H3GA0020849 and ASGA0032595) contained four annotated genes, namely, *t-complex 11, testis-specific* (*TCP11*); *signal peptide, CUB domain, EGF-like 3* (*SCUE3*); *peroxisome proliferator-activated receptor delta* (*PPARD*); and *FK506 binding protein 5* (*FKBP5*).

## Discussion

We performed a GWAS for six porcine limb bone lengths and identified significant SNPs on the long arm of SSC7 between 31.24 and 43.17 Mb. A single QTL can influence multiple traits [4]. All significant SNPs were clustered in the same region, and the high correlation (0.62 to 0.78) among the six traits suggested that a QTL with a pleiotropic effect on limb bone lengths was located in the 11.93 Mb region identified on SSC7q. To our knowledge, this is the first time that a genome-wide association of SNPs with HIPL is reported in pigs. These SNPs, which were also associated with the five other traits (FL, HL, SL, TL, and UL), were located within known QTL regions. Previously, a 108.89 Mb region (between 12.19 and 121.08 Mb) on SSC7 was reported to harbor a QTL that influenced limb bone length [9]. Our results narrow down this region to 11.93 Mb.

The additional significant SNPs found at the distal end of the SSC7q may result from the large linkage disequilibrium that exists in the F2 design population [18]. Although the GWAS based on an F2 population design effectively narrowed down the QTL region, the large linkage disequilibrium present in this population is a limitation that can result in a larger linkage region than that observed using natural populations in livestock [19] or humans [20]. Linkage disequilibrium analysis was performed using all 233 chromosome-wide significant SNPs. The fact that the SNPs at the distal end of SSC7q and other significant SNPs were not in LD [See Additional file 7: Figure S3] indicates that there was no obvious error in the genome assembly and the second peak in this region might be caused by a second QTL.

To overcome the above limitation, post-GWAS analysis often includes haplotype analysis, which is a reliable method to identify the minimum segment that contains the causal gene [8]. Three haplotype blocks, respectively 376 kb, 492 kb, and 495 kb long, were detected. Fifteen annotated genes were near or contained the significant SNPs of these three haplotype blocks. According to the mouse gene expression data in MGI (http://www.informatics.jax.org/), the genes *GRM4*, *HMGA1*, *RPS10*, *SPDEF*, *SCUBE3*, *PPARD* and *FKBP5* are expressed in mouse limbs. However, *GRM4*, *RPS10*, *SPDEF*, *SCUBE3*, and *FKBP5* are, respectively, involved in the regulation of neural stem cell differentiation [21], mediation of mammary luminal epithelial lineage-specific gene expression [22], association with diamond-Blackfan anemia [23], regulation of early lung cancer angiogenesis and metastatic progression [24], and regulation of the immunosuppressive function of myeloid-derived suppressor cells [25]. None of these genes have been reported to be associated with bone growth and, thus, cannot be considered as good candidates for limb bone length. The remaining genes, *HMGA1* and *PPARD,* are involved in chondrocyte cell growth and differentiation. HMGA1 is ubiquitous in all cells of higher eukaryotes [26] and is known to have a biological role in cell growth and differentiation [27]. Silencing *HMGA1* expression in invasive, aggressive cancer cells dramatically arrests cell growth and blocks their oncogenic properties, including proliferation, migration, invasion, and orthotopic tumorigenesis [28]. It was also shown that in vitro culture of porcine chondrocytes in the presence of HMGA1 increased their proliferation, which suggests that it could be a promising approach to enhance cartilage tissue repair and growth [29]. Furthermore, *HMGA1* influences the expression of *insulin growth factor-binding protein* (*IGFBP*) and thus acts as a modulator of *insulin-like growth factor I* (*IGF-I*) activity [30], a gene which has been reported to be involved in bone growth, tibia length, body height, and body size in both humans and animals [31-35]. In addition, human GWAS have suggested that *HMGA1* may be a good candidate for anthropometric traits. In Western European populations, GWAS revealed that SNP rs6918981 that is located near the *HMGA1* gene contributed to height variation (*P* < 10^−7^) [36]. Moreover, another SNP (rs1776897) located within the *HMGA1* gene has been reported to have genome-wide associations with human height (*P* = 1.6E-08) and hip axis length (*P* = 0.005) [37].

Regarding the *PPARD* gene, Duan et al. [38] reported that its activation can inhibit chondrocyte differentiation and growth in pigs. Several reports have identified a *PPARD* variant associated with limb bone length, which indicates that it could be a good candidate for human height. A GWAS that included 183 727 individuals showed that the *PPARD* gene was associated with human height [39]. Another GWAS study on individuals of African ancestry showed that an SNP (rs9470004) that is located in *PPARD* was associated with adult height (*P* = 1.0E-11) [40]. Therefore, both *HMGA1* and *PPARD* can be considered as good candidates for limb bone length and should be studied further.

## Conclusions

As for the human studies described above, our GWAS on a pig population revealed that the *HMGA1* and *PPARD* genes displayed significant association with porcine limb bone length. These results confirm that pig is an appropriate large animal model for human height and limb length research. We conclude that *HMGA1* and *PPARD* are relevant biological candidates for limb bone length in pigs. Further analyses of these genes based on additional genetic, functional, and computational studies are expected to provide novel insights into the genetic mechanisms responsible for limb bone length in different pig breeds and also perhaps for human height.

## Additional files

Additional file 1: Table S1. Distribution of SNPs after quality control and average distances between SNPs on each chromosome. After quality control, a total of 48 238 SNPs and 564 F2 individuals were used for the GWAS. According to the genome information of *sus scrofa* Build 10.2, the selected SNPs were distributed over 18 autosomes and the X chromosome. Additional file 2: Table S2. Genome-wide significant SNPs on SSC7 associated with limb bone lengths. Thirty-nine SNPs that are contained in a 11.93 Mb (between 133.96 and 134.68 Mb) region at the long arm of SSC7 (i.e. SSC7q) were significantly associated (*P* < 2.07E-08) with limb bone lengths. Additional file 3: Table S3. Chromosome-wide significant SNPs with limb bone length in the distal region of SSC7. Eleven SNPs that are contained in a 0.72 Mb (between 133.96 and 134.68 Mb) region at the distal end of SSC7q were significantly associated (*P* < 6.16E-05) with limb bone length. Additional file 4: Figure S1. Manhattan plots obtained from the conditioned analysis. After conditioned analysis, no significant SNP was detected on SSC7. However, four SNPs, one on SSC2 and three on SSC4, showed chromosome-wide associations with SL and HL, respectively. The Manhattan plots are shown for CW, HTW and LUW and chromosome-wide significant SNPs for CL, FW and HW. Additional file 5: Figure S2. Q-Q plots obtained from the conditioned analysis. After conditioned analysis, the results of the Q-Q plot showed an obvious deviation between the real and expected data and indicated that associations on SSC2 and SSC4 are statistically significant. Additional file 6: Table S4. SNPs significantly associated with limb bone lengths after conditioned analysis. After conditioned analysis, four SNPs, one on SSC2 and three on SSC4, showed chromosome-wide associations with SL and HL, respectively. Additional file 7: Figure S3. Linkage disequilibrium analysis of 233 chromosome-wide significant SNPs. Using 233 chromosome-wide significant SNPs, linkage disequilibrium analysis was performed and identified 19 haplotype blocks (solid lines).
