# Supplementary material for: A genome-wide association study of limb bone length using a Large White × Minzhu intercross population
Source: Genet Sel Evol. 2014 Nov 4;46(1):56. doi: 10.1186/s12711-014-0056-6 (PMC4219012; doi:10.1186/s12711-014-0056-6)
Supplement: Additional file 1: Table S1. — Distribution of SNPs after quality control and average distances between SNPs on each chromosome. After quality control, a total of 48 238 SNPs and 564 F2 individuals were used for the GWAS. According to the genome information of sus scrofa Build 10.2, the selected SNPs were distributed over 18 autosomes and the X chromosome. [file 12711_2014_56_MOESM1_ESM.doc]

**Additional file 1: Table S1** Distribution of SNPs after quality control and the average distances between SNPs on each chromosome

| **Chromosome** | **No. SNPs** | **Average distance(kb)** |
| --- | --- | --- |
| 1 | 5323 | 59.16 |
| 2 | 2759 | 58.82 |
| 3 | 2228 | 64.69 |
| 4 | 2937 | 48.81 |
| 5 | 1971 | 56.43 |
| 6 | 2620 | 60.02 |
| 7 | 2779 | 48.45 |
| 8 | 2308 | 63.93 |
| 9 | 2668 | 57.52 |
| 10 | 1496 | 52.62 |
| 11 | 1574 | 55.67 |
| 12 | 1308 | 48.49 |
| 13 | 3384 | 64.57 |
| 14 | 3136 | 48.99 |
| 15 | 2361 | 66.68 |
| 16 | 1565 | 55.50 |
| 17 | 1381 | 50.09 |
| 18 | 1133 | 53.74 |
| X | 733 | 195.70 |
| 0^1^ | 4574 |  |
| Total | 48238 |  |

^1^These SNPs are not assigned to any chromosomes.
