# Supplementary material for: A genome-wide association study of limb bone length using a Large White × Minzhu intercross population
Source: Genet Sel Evol. 2014 Nov 4;46(1):56. doi: 10.1186/s12711-014-0056-6 (PMC4219012; doi:10.1186/s12711-014-0056-6)
Supplement: Additional file 2: Table S2. — Genome-wide significant SNPs on SSC7 associated with limb bone lengths. Thirty-nine SNPs that are contained in a 11.93 Mb (between 133.96 and 134.68 Mb) region at the long arm of SSC7 (i.e. SSC7q) were significantly associated (P < 2.07E-08) with limb bone lengths. [file 12711_2014_56_MOESM2_ESM.doc]

**Additional file 2: Table S2 Genome-wide significant SNPs on SSC7 associated with limb bone lengths^1^**

| **SNP** | **Position** | **Nearest gene** | **Distance**  **(bp)** | **P-value (TL)** | **Var(%)** | **P-value (HL)** | **Var(%)** | **P-value (FL)** | **Var(%)** | **P-value (UL)** | **Var(%)** | **P-value (HIPL)** | **Var(%)** | **P-value (SL)** | **Var(%)** |
| --- | --- | --- | --- | --- | --- | --- | --- | --- | --- | --- | --- | --- | --- | --- | --- |
| ALGA0039921 | 31237418 | *LRRC1* | 65126 | 6.28E-09 | 41.61 | 1.35E-08 | 48.52 |  |  | 1.08E-08 | 47.88 |  |  |  |  |
| DRGA0007448 | 31628039 | *KLHL31* | 28731 | 8.19E-09 | 41.52 | 1.50E-08 | 48.66 |  |  | 1.05E-08 | 48.02 |  |  |  |  |
| ASGA0032302 | 32957768 | *PRIM2* | within | 7.85E-09 | 41.75 | 7.06E-09 | 49.59 | 1.59E-08 | 36.55 | 5.73E-09 | 48.65 |  |  |  |  |
| ASGA0032313 | 33086096 | *RAB23* | 5782 | 8.84E-09 | 41.10 | 6.61E-09 | 49.39 | 1.59E-08 | 36.27 | 5.38E-09 | 48.46 |  |  |  |  |
| ALGA0040120 | 33740960 | *DST* | within | 4.92E-09 | 43.54 | 1.27E-08 | 51.64 | 1.17E-08 | 38.10 |  |  |  |  |  |  |
| H3GA0020692 | 33790291 | *DST* | 38479 | 1.60E-09 | 45.03 | 1.22E-08 | 51.70 | 1.04E-08 | 38.72 |  |  |  |  |  |  |
| MARC0079017 | 33876748 | *COL9A3* | 42044 | 2.07E-08 | 43.73 |  |  |  |  |  |  |  |  |  |  |
| ALGA0040148 | 33991469 | *COL9A3* | within | 9.51E-10 | 46.50 | 2.53E-09 | 54.07 | 7.55E-10 | 42.55 | 8.51E-09 | 52.33 |  |  |  |  |
| H3GA0020739 | 34556148 | *MNF1* | 18523 | 5.29E-11 | 48.35 | 1.90E-10 | 55.42 | 2.20E-11 | 45.97 | 3.22E-10 | 54.64 | 1.95E-08 | 50.24 | 7.83E-09 | 36.45 |
| H3GA0020765 | 34755602 | *MLN* | 82751 | 1.67E-11 | 50.41 | 5.23E-11 | 57.63 | 1.12E-11 | 47.41 | 1.20E-10 | 57.19 | 7.40E-09 | 51.67 | 2.63E-09 | 38.08 |
| MARC0058766 | 34803564 | *GRM4* | 35677 | 2.06E-11 | 50.22 | 6.43E-11 | 57.50 | 1.30E-11 | 47.23 | 1.52E-10 | 57.05 | 9.54E-09 | 51.20 | 4.73E-09 | 37.97 |
| ALGA0040260 | 35002839 | *HMGA1* | 12750 | 1.53E-09 | 39.86 | 1.11E-09 | 48.13 | 1.45E-09 | 35.43 | 2.95E-09 | 45.40 | 1.66E-08 | 45.22 |  |  |
| ALGA0040263 | 35017672 | *NUDT3* | within | 1.53E-09 | 39.86 | 1.11E-09 | 48.13 | 1.45E-09 | 35.43 | 2.95E-09 | 45.40 | 1.66E-08 | 45.22 |  |  |
| ASGA0032536 | 35150544 | *RPS10* | 33110 | 1.53E-09 | 39.92 | 1.11E-09 | 48.15 | 1.45E-09 | 35.40 | 2.95E-09 | 45.36 | 1.66E-08 | 45.22 |  |  |
| MARC0033464 | 35177641 | *C7H6orf106* | within | 5.30E-12 | 51.97 | 2.99E-11 | 57.89 | 8.94E-12 | 47.93 | 6.23E-11 | 57.80 | 4.07E-09 | 52.28 | 1.90E-09 | 37.50 |
| ASGA0032526 | 35251345 | *PACSIN1* | 7190 | 1.53E-09 | 39.92 | 1.11E-09 | 48.17 | 1.45E-09 | 35.50 | 2.95E-09 | 45.36 | 1.66E-08 | 45.22 |  |  |
| H3GA0020824 | 35332373 | *C6ORF106* | 30460 | 3.98E-09 | 39.20 | 3.20E-09 | 47.42 | 2.06E-08 | 33.06 | 6.20E-09 | 44.85 |  |  |  |  |
| ASGA0032549 | 35356274 | *C6ORF106* | 6561 | 3.98E-09 | 39.20 | 3.20E-09 | 47.42 | 2.06E-08 | 33.15 | 6.20E-09 | 44.85 |  |  |  |  |
| ASGA0032562 | 35530333 | *UHRF1BP1* | within | 1.07E-09 | 39.82 | 1.11E-09 | 47.90 | 6.12E-09 | 33.92 | 2.98E-09 | 44.97 | 1.42E-08 | 45.50 |  |  |
| INRA0024805 | 35579961 | *UHRF1BP1* | 41454 | 1.01E-09 | 40.05 | 9.91E-10 | 48.49 | 5.59E-09 | 34.31 | 2.43E-09 | 45.71 | 1.40E-08 | 45.87 |  |  |
| ASGA0032571 | 35709335 | *ANKS1A* | within | 1.07E-09 | 39.82 | 1.11E-09 | 47.90 | 6.12E-09 | 33.92 | 2.98E-09 | 44.97 | 1.42E-08 | 45.50 |  |  |
| M1GA0010006 | 35880196 | *SCUBE3* | 5106 | 2.25E-09 | 39.43 | 1.39E-09 | 48.21 | 6.24E-09 | 34.15 | 3.93E-09 | 45.17 |  |  |  |  |
| MARC0039836 | 35935629 | *SCUBE3* | 60539 | 5.41E-11 | 47.62 | 1.89E-10 | 55.34 | 1.33E-10 | 42.95 | 4.53E-10 | 53.92 | 2.06E-08 | 50.08 | 6.68E-09 | 35.85 |
| H3GA0020842 | 35959385 | *SCUBE3* | 84295 | 1.32E-09 | 46.31 | 2.91E-09 | 54.15 | 1.83E-09 | 41.85 | 4.47E-09 | 53.39 |  |  |  |  |
| H3GA0020849 | 36004578 | *SCUBE3* | 137056 | 5.46E-11 | 47.62 | 1.98E-10 | 55.29 | 1.35E-10 | 42.85 | 4.56E-10 | 53.92 |  |  | 8.64E-09 | 35.53 |
| ASGA0032583 | 36169891 | *PPARD* | within | 4.72E-09 | 38.84 | 2.86E-09 | 47.67 | 1.25E-08 | 33.60 | 7.21E-09 | 44.60 |  |  |  |  |
| H3GA0020846 | 36202231 | *PPARD* | within | 4.72E-09 | 38.84 | 2.86E-09 | 47.67 | 1.25E-08 | 33.60 | 7.21E-09 | 44.60 |  |  |  |  |
| INRA0024809 | 36329680 | *FKBP5* | 40890 | 5.89E-09 | 38.63 | 3.33E-09 | 47.54 | 1.58E-08 | 33.37 | 8.77E-09 | 44.33 |  |  |  |  |
| ASGA0032595 | 36497507 | *FKBP5* | 18996 | 4.51E-11 | 58.66 | 2.45E-10 | 55.28 | 2.57E-10 | 42.99 | 5.03E-10 | 54.42 |  |  |  |  |
| ALGA0040331 | 36684494 | *SLC26A8* | within | 5.89E-09 | 38.63 | 3.33E-09 | 47.54 | 1.58E-08 | 33.37 | 8.77E-09 | 44.33 |  |  |  |  |
| ALGA0040423 | 37731099 | *TMEM217* | within | 8.33E-09 | 38.30 | 1.38E-08 | 45.67 |  |  |  |  |  |  |  |  |
| DIAS0000130 | 39089506 | *BTBD9* | within | 8.45E-09 | 38.08 | 2.05E-08 | 44.85 |  |  |  |  |  |  |  |  |
| ALGA0040570 | 39627760 | *GLP1R* | 4327 | 8.08E-09 | 38.08 | 1.95E-08 | 44.85 |  |  |  |  |  |  |  |  |
| ASGA0032851 | 39985401 | *KIF6* | within | 9.86E-09 | 37.81 |  |  |  |  |  |  |  |  |  |  |
| MARC0051108 | 40265147 | *DAAM2* | 99976 | 1.22E-08 | 37.65 |  |  |  |  |  |  |  |  |  |  |
| INRA0025056 | 40524179 | *MOCS1* | 72980 | 7.27E-09 | 43.67 | 1.99E-08 | 49.06 |  |  |  |  |  |  |  |  |
| ASGA0032963 | 40847468 | *LRFN2* | 29599 | 6.36E-09 | 43.40 | 1.66E-08 | 48.56 | 1.79E-08 | 37.45 |  |  |  |  |  |  |
| ALGA0040717 | 41004531 | *LRFN2* | 51620 | 5.97E-09 | 43.26 | 1.43E-08 | 48.66 | 1.86E-08 | 37.45 |  |  |  |  |  |  |
| ALGA0040856 | 43166101 | *UBR2* | within | 9.57E-09 | 44.67 |  |  |  |  |  |  |  |  |  |  |

^1^FL, femur length; HL, humerus length; HIPL, hipbone length; SL, scapula length; TL, tibia length; UL, ulna length. Var(%) means phenotypic variation explained by the SNP.
