# Supplementary material for: A genome-wide association study of limb bone length using a Large White × Minzhu intercross population
Source: Genet Sel Evol. 2014 Nov 4;46(1):56. doi: 10.1186/s12711-014-0056-6 (PMC4219012; doi:10.1186/s12711-014-0056-6)
Supplement: Additional file 3: Table S3. — Chromosome-wide significant SNPs with limb bone length in the distal region of SSC7. Eleven SNPs that are contained in a 0.72 Mb (between 133.96 and 134.68 Mb) region at the distal end of SSC7q were significantly associated (P < 6.16E-05) with limb bone length. [file 12711_2014_56_MOESM3_ESM.doc]

**Additional file 3: Table S3** Chromosome-wide significant SNPs with limb bone length in the distal region of SSC7^1^

| **SNP** | **Position** | **Nearest gene** | **Distance**  **(bp)** | ***P*-value (UL)** | ***P*-value (HL)** | ***P*-value (FL)** | ***P*-value (TL)** | ***P*-value (SL)** | ***P*-value (HIPL)** |
| --- | --- | --- | --- | --- | --- | --- | --- | --- | --- |
| ASGA0037322 | 133962789 | *EFHC1* | 3984 | 5.15E-05 | 2.78E-05 | 3.84E-05 | 6.06E-05 |  |  |
| ASGA0037335 | 134077383 | *TRAM2* | within | 1.3E-05 | 8.05E-06 | 1.14E-05 | 5.69E-05 | 6.01E-05 |  |
| MARC0060185 | 134109770 | *TRAM2* | within | 4.45E-05 | 2.35E-05 | 2.84E-05 |  |  |  |
| MARC0039911 | 134128276 | *TRAM2* | within | 3.91E-06 | 5.75E-06 | 1.79E-06 | 1.54E-05 | 2.69E-05 |  |
| M1GA0011538 | 134236731 | *LOC100512176* | 17744 | 2.16E-06 | 2.58E-06 | 6.01E-07 | 6.45E-06 | 3.86E-06 | 3.06E-05 |
| MARC0093860 | 134275238 | *LOC100511647* | 59 | 2.87E-06 | 4.05E-06 | 1.38E-06 | 1.19E-05 | 1.99E-05 |  |
| MARC0060950 | 134313767 | *GSTA1* | 1734 | 3.17E-06 | 4.70E-06 | 1.31E-06 | 1.23E-05 | 1.49E-05 |  |
| MARC0058228 | 134388813 | *GSTA4* | within | 4.05E-05 | 4.06E-05 | 1.38E-05 |  |  |  |
| ALGA0045987 | 134405082 | *ICK* | 1043 | 3.98E-05 | 3.81E-05 | 1.36E-05 |  |  |  |
| H3GA0023987 | 134562880 | *GCM1* | 46213 | 4.45E-05 | 4.17E-05 | 1.51E-05 |  |  |  |
| ALGA0046005 | 134683639 | *LOC102166388* | 10543 | 3.05E-06 | 4.77E-06 | 1.17E-06 | 1.55E-05 | 9.68E-06 | 6.13E-05 |

^1^Chromosome-wide significant threshold on SSC7 is 6.16E-05. SL, scapula length; HL, humerus length; UL, ulna length; HIPL, hipbone length; FL, femur length; TL, tibia length.
