# Supplementary figures and images for: A genome-wide association study of limb bone length using a Large White × Minzhu intercross population
Source: Genet Sel Evol. 2014 Nov 4;46(1):56. doi: 10.1186/s12711-014-0056-6 (PMC4219012; doi:10.1186/s12711-014-0056-6)

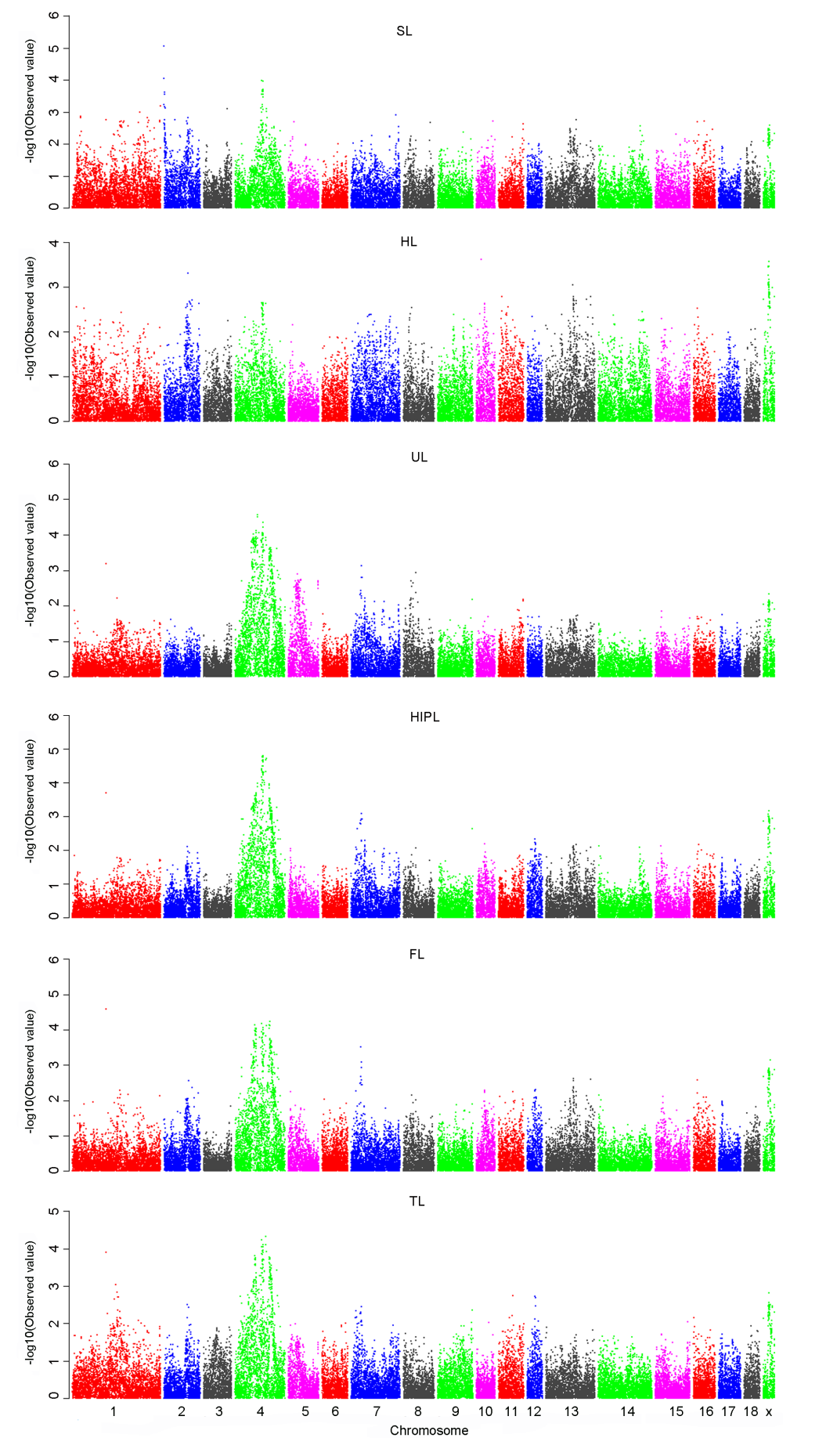


Additional file 4: Figure S1 Manhattan plots obtained from the conditioned analysis.

Supplement: Additional file 4: Figure S1. — Manhattan plots obtained from the conditioned analysis. After conditioned analysis, no significant SNP was detected on SSC7. However, four SNPs, one on SSC2 and three on SSC4, showed chromosome-wide associations with SL and HL, respectively. The Manhattan plots are shown for CW, HTW and LUW and chromosome-wide significant SNPs for CL, FW and HW. [file 12711_2014_56_MOESM4_ESM.doc]

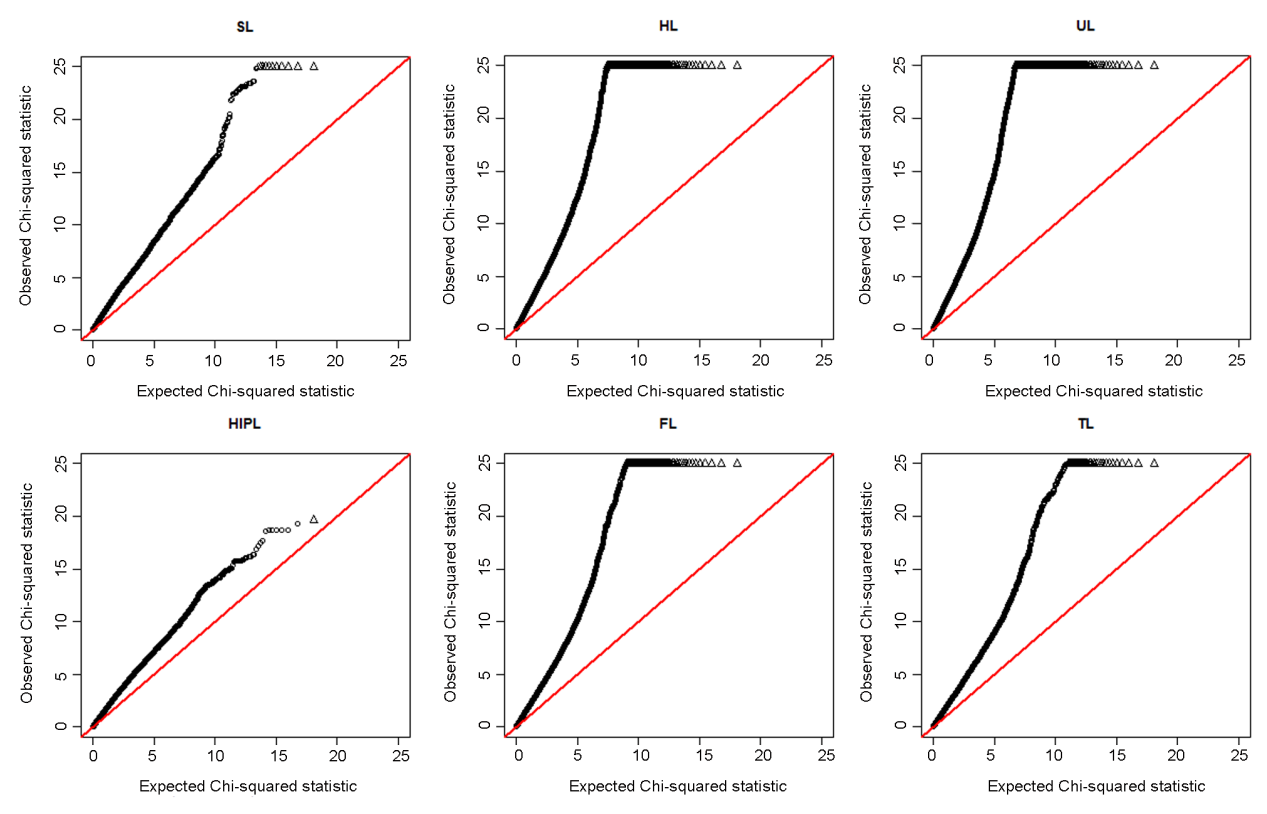


Additional file 5: Figure S2 Q-Q plots obtained from the conditioned analysis.

Supplement: Additional file 5: Figure S2. — Q-Q plots obtained from the conditioned analysis. After conditioned analysis, the results of the Q-Q plot showed an obvious deviation between the real and expected data and indicated that associations on SSC2 and SSC4 are statistically significant. [file 12711_2014_56_MOESM5_ESM.doc]
