# Supplementary material for: A genome-wide association study of limb bone length using a Large White × Minzhu intercross population
Source: Genet Sel Evol. 2014 Nov 4;46(1):56. doi: 10.1186/s12711-014-0056-6 (PMC4219012; doi:10.1186/s12711-014-0056-6)
Supplement: Additional file 6: Table S4. — SNPs significantly associated with limb bone lengths after conditioned analysis. After conditioned analysis, four SNPs, one on SSC2 and three on SSC4, showed chromosome-wide associations with SL and HL, respectively. [file 12711_2014_56_MOESM6_ESM.doc]

**Additional file 6: Table S4** SNPs significantly associated with limb bone lengths after conditioned analysis ^1^

| **Trait** | **SNP** | **Chr.** | **Position (bp)** | **Nearest gene** | **Distance (bp)** | **P-value** |
| --- | --- | --- | --- | --- | --- | --- |
| **SL** | MARC0053324 | 2 | 489542 | *CARS* | 5193 | 8.70E-06 |
| **HL** | MARC0012321 | 4 | 84599167 | *OPRK1* | 102246 | 1.37E-05 |
|  | SIRI0000865 | 4 | 85105668 | *ST18* | within | 1.68E-05 |
|  | ASGA0020433 | 4 | 85250559 | *PCMTD1* | 116822 | 1.61E-05 |

^1^Chromosome-wide significant thresholds on SSC2 and SSC4 are 2.36E-05 and 1.72E-05, respectively. SL, scapula length; HL, humerus length; UL, ulna length; HIPL, hipbone length; FL, femur length; TL, tibia length.
