# Supplementary material for: A genome-wide association study of limb bone length using a Large White × Minzhu intercross population
Source: Genet Sel Evol. 2014 Nov 4;46(1):56. doi: 10.1186/s12711-014-0056-6 (PMC4219012; doi:10.1186/s12711-014-0056-6)
Supplement: Additional file 7: Figure S3. — Linkage disequilibrium analysis of 233 chromosome-wide significant SNPs. Using 233 chromosome-wide significant SNPs, linkage disequilibrium analysis was performed and identified 19 haplotype blocks (solid lines). [file 12711_2014_56_MOESM7_ESM.doc]

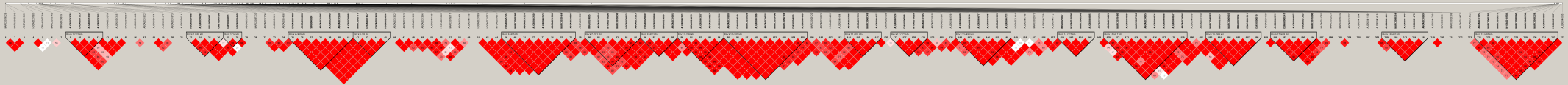


Additional file 7: Figure S3 Linkage disequilibrium analysis of 233 chromosome-wide significant SNPs
